# Effect of Benzothiazole based conjugates in causing apoptosis by Regulating p53, PTEN and MAP Kinase proteins affecting miR-195a and miR-101-1

**DOI:** 10.1186/1475-2867-11-36

**Published:** 2011-10-28

**Authors:** SNCVL Pushpavalli, M Janaki Ramaiah, Ch Srinivas, Debasmita Mukhopadhya, JL Aditya, Ravindra M Kumbhare, Utpal Bhadra, Manika Pal Bhadra

**Affiliations:** 1 Division of Chemical Biology, Indian Institute of Chemical Technology, Tarnaka, Hyderabad-500607, India; 2 Division of Functional Genomics and Gene silencing, Centre for Cellular and Molecular Biology, Tarnaka, Hyderabad-500007, India; 3 Division of Fluoro organics, Indian Institute of Chemical Technology, Tarnaka, Hyderabad-500607, India

## Abstract

**Background:**

Hepatocellular carcinoma (HCC) accounts for majority of liver cancers and is the leading cause of cancer related death in Asia. Like any other cancer, HCC develops when there is a mutation to the cellular machinery that causes the cell to replicate at a higher rate and results in the loss of apoptosis. Therefore, a delicate balance between the expression of various genes involved in proliferation and apoptosis decide the ultimate fate of the cell to undergo rapid proliferation (cancer) or cell death.

**Results:**

The benzothiazole based compounds exhibited effective cytotoxicity at 4 μM concentration and have shown G1 cell cycle arrest with decrease in levels of G1 cell cycle proteins such as cyclin D1 and Skp2. Involvement of tumour suppressor proteins such as PTEN and p53 was studied. Interestingly these compounds displayed decrease in the phosphorylated forms of AKT, p38 MAPK and ERK1/2 which play a vital role in cell proliferation. Compounds have exhibited strong and significant effect on the expression of micro RNAs such as miR-195a & miR-101-1 which regulate hepatic cell proliferation.

**Conclusions:**

The cell cycle arrest and apoptotic inducing nature of these compounds was revealed by FACS, BrdU cell proliferation and tunel assays. Compounds affected both tumour suppressor proteins as well as proteins that are involved in active cell proliferation. Micro RNAs whose target is Cyclin D1 such as miR-195a and miR-101-1 that is required for growth of hepatoma cells was drastically affected. These compounds caused apoptosis by activating caspase-3 and PARP.

## Background

Hepato cellular carcinoma (HCC) is one among the most prevalent and lethal cancers in humans [1] and is the fifth leading cause of cancer across the world. Majority of the patients with HCC do not survive because of the frequent recurrence of tumour and metastasis. With the recent developments in the field of molecular oncology, researchers have focussed their efforts towards the control of HCC [2,3]. The key signalling molecules that are frequently activated and involved in human hepatoma are Ras, ERK (extracellular signal regulated kinase) and AKT [4]. Phosphotidyl inositol-3-kinase (PI3K/AKT) constitute an upstream important pathway that regulates the processes of cell growth, metabolism, proliferation and apoptosis [5]. Mitogen activated protein kinases (MAPKs) such as ERK1/2, JNK (c-Jun NH2-terminal kinase), and p38 MAPK belongs to serine/threonine kinase protein family that mediates the intracellular signals in response to various extracellular stimuli. Among these, ERK 1/2 mainly functions in controlling cell division and is considered to provide survival signals that can lead to rapid rate of cell proliferation [6]. Hence inhibitors of ERK are being explored for their anti-cancer properties.

Earlier studies have indicated that HCC may be the cumulative result of inactivation of tumour suppressor gene and activation of multiple oncogenes [7]. PTEN and p53 mainly functions as tumour suppressors and regulate the process of apoptosis [8]. Apoptosis is a programmed cell death which is characterized by events like cytoplasmic shrinkage, chromatin condensation, plasma membrane blebbing, exposure of phosphotidyl serine (PS) of the cell membrane from interior to exterior [9]. Resistance to apoptosis was reported to be a significant factor for hepato-carcinogenesis [10]. Thus compounds having apoptotic inducing nature will certainly play a crucial role in preventing the rapid cell proliferation and ultimately hepatocarcinoma.MicroRNAs (miRNAs) are a class of phylogenetically conserved short RNAs that suppress protein expression through base-pairing with the 3'-untranslated region (3'-UTR) of target mRNA. MicroRNAs are known to play important roles in cell growth, differentiation, proliferation and apoptosis. MiRNAs associated with tumourigenesis act as either tumour suppressors or oncogenes [11] and are used as potential diagnostic or therapeutic targets in cancer treatment [12]. Although aberrant microRNA (miRNA) expression has been observed in different types of cancer, their pathophysiological role and their relevance to tumourigenesis are still largely unknown. Drastic downregulation of miR-195a, miR-101-1 has been observed in hepatoma (metastasis) [13,14].

**Figure 1:**
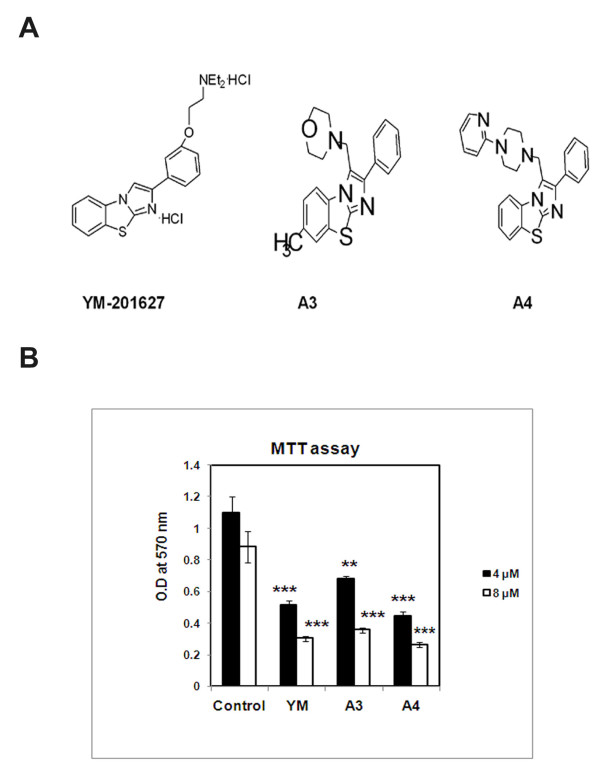
**Effect of benzothiazole based compounds on cell viability**. (**A**) Chemical structures of YM, A3 and A4 compounds. **(B) **The HepG2 cells were subjected to compound treatments YM, A3, A4 at 4 and 8 μM concentrations for 24h. MTT assay was carried out and the O.D a 570 nm values are recorded which represents viable cells left over after compound treatment. Each experiment was conducted three times. The p values are derived in compound treated cells and are statistically significant when compared to control untreated cells. ** indicates p < 0.05 and *** indicates p < 0.001.

YM-201627 (**YM**), a 2-arylimidazo [2,1-b][1,3]benzothiazole derivative, was identified as an inhibitor of VEGF-stimulated angiogenesis and is thought to play a critical role in tumour growth and metastasis [15,16]. To increase the cytotoxicity of hepatic cancer cells we have synthesised two analogs 3-(morpholinomethyl)-2-phenyl-7-methyl-imidazo[2,1-b]benzothiazole (**A3**) and 3-(4-(2-pyridinyl)piperazinomethyl)-2-phenylimidazo[2,1-b]benzothiazole (**A4**) (Figure 1A).

Earlier studies have demonstrated the anti-angiogenesis nature of YM compound in HUVEC cells but a detailed study regarding the pathway has so far not been carried out. In the present study we have focussed mainly on the pathway targeted by benzothiazole conjugates which results in effective cytotoxicity in hepatoma cells by modulating the expression of both oncogenic and tumour suppressor proteins.

## Results

### Benzothiazole based compounds induce Cytotoxicity

**Figure 2:**
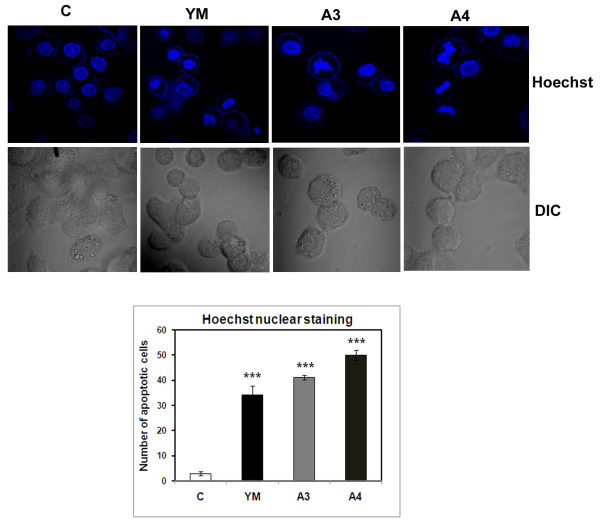
**Immunofluorescence studies to observe apoptotic related morphological changes in cells treated with benzothiazole based compounds**. HepG2 cells were grown in DMEM medium with 10% FBS for over night time period. Conjugates (YM, A3 and A4) were treated at 4μM for 24 h time period. YM is the standard drug. The cells were then washed, fixed in 4% paraformaldehyde and stained with nuclear dye Hoechst. The slides were then examined by fluorescence microscopy and photographed. Cells with signs of apoptosis (blebbing) were observed in YM, A3, and A4 compound treated cells when compared with untreated cells (C). The p values are derived in compound treated cells and are statistically significant when compared to control. *** indicates p < 0.001.

**Figure 3:**
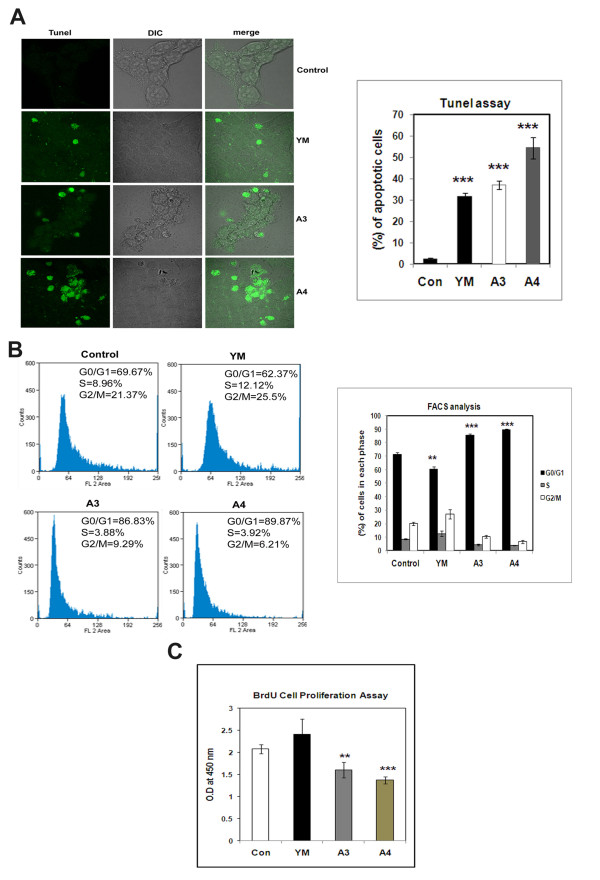
**Effect of Benzothiazole based compounds on DNA fragmentation and cell cycle in HepG2 cells**. **(A) **HepG2 cells were treated with compounds YM, A3 and A4 at a concentration of 4 μM for a time period of 24 h. The standard compound used here in this study is YM. The fragmented DNA was bind to antibody conjugated to FITC. The fluorescence was detected and imaged using confocal microscope. The results obtained were the mean of three independent experiments and were found to be statistically significant with p-value <0.001. **(B) **FACS analysis was conducted after treatment of HepG2 cells with compounds YM, A3, A4 at a final concentration of 4 μM for 24 h.The standard compound used here in this study YM. YM has shown G2/M cell cycle arrest. A3 and A4 compounds have shown clear G1 cell cycle arrest. Each experiment was repeated three times and the results were statistically significant with p-value <0.01 for YM, <0.001 for A3 and A4. **(C) **The HepG2 cells were treated with YM, A3 and A4 compounds for 24 h. The cells were subjected to BrdU incorporation where in proliferating cells from G1 to S-phase of cell cycle accumulate more BrdU than those of G1 arrested cells. Here the BrdU antibody was added and was detected by secondary HRP antibody. O.D at 450 nm will give the amount of BrdU incorporated. The p values are derived in compound treated cells when compared to control cells and are statistically significant with the p-value of <0.01 for A3 and <0.001 for A4.

HepG2 cells (human hepatocarcinoma) were treated with benzothiazole based compounds YM, A3 and A4 for 24 h using YM as the standard benzothiazole compound in the present study. At 4 μM concentration, these compounds caused 50% cell death and upon increase of concentration to 8 μM, we observed 25% more increase in cell death (Figure 1B). To study the apoptosis inducing nature of these compounds, the cells were stained with Hoechst nuclear staining dye wherein, apoptotic cells have shown a typical blebbing nature. Nearly 50% cells undergo apoptosis in both A3 and A4 treated cells (Figure 2) with clear DNA fragmentation which is the characteristic feature of apoptosis as observed by performing Tunel assay (Terminal deoxynucleotidyl transferase dUTP nick end labelling) a common method for detecting DNA fragmentation that results from apoptotic signalling cascades (Figure 3A).

### Effect of Benzothiazole based compounds on Cell Cycle

In order to understand whether benzothiazole based compounds (A3 and A4) have any regulatory role on cell cycle, FACS analysis was conducted. HepG2 cells were treated with compounds YM, A3 and A4 for 24 h. Treatment of cells with compounds A3 and A4 caused G1 cell cycle arrest, whereas YM the standard drug has caused G2/M cell cycle arrest. The percentage of cells in the G1 phase was 87 and 90 in case of A3 and A4 compound treated cells respectively. YM has shown 25% cells in G2/M phase and control untreated cells have shown 69% and 21% in G1 and G2/M phases respectively (Figure 3B). The G1 cell cycle arrest caused by A3 and A4 compounds were further confirmed by BrdU cell proliferation assay which reveals whether the cells are arrested in G1 or progressing to S phase of cell cycle. Here the control and YM treated cells have not shown any change in the O.D values whereas A3 and A4 treated cells have shown reduction, confirming the G1 phase arrest caused by these compounds (Figure 3C).

### Effect of Benzothiazole based compounds on cell cycle regulatory proteins

**Figure 4:**
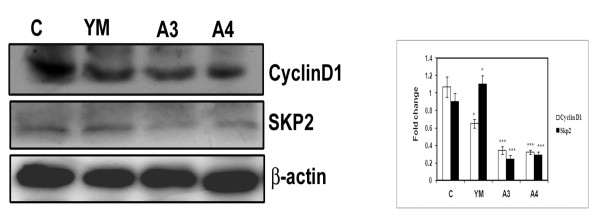
**Effect of benzothiazole based compounds on cell cycle regulatory proteins Cyclin D1, Skp2 in HepG2 cells**. HepG2 cells treated at 80% confluency with YM, A3 and A4 at 4 μM concentration for 24 h. The total cell lysates were isolated and analysed by immunoblotting with anti-CyclinD1, anti-Skp2. YM is the positive control. β-actin was used as loading control. Western blots were representative of three independent experiments. C represents control untreated cells. The molecular weight of CyclinD1 and Skp2 were **35**, **47 **KDa. Each experiment was repeated three times and densitometric analysis was done. Results were statistically significant with p-value <0.001 for A3 and A4 when compared to control.

Cyclin D1 and Skp2 are the critical regulators of G1 to S phase transition of cell cycle. In order to understand the effect of these compounds on cell cycle regulatory proteins, HepG2 cells were treated with compounds YM, A3 and A4 for 24 h. The cell lysates were subjected to western blot analysis using antibodies against Cyclin D1 and Skp2. A drastic reduction in the protein levels of Cyclin D1 and Skp2 in A3 and A4 treated cells compared to untreated control but not in YM indicated the occurrence of G1 cell cycle arrest (Figure 4).

### Benzothiazole based compounds activate tumour suppressor proteins p53 and PTEN

P53 and PTEN are tumour suppressor proteins that control the apoptotic event. p53 function is crucial in multicellular organisms, where it regulates cell cycle and thus functions as a tumour suppressor that is involved in preventing cancer. PTEN is one of the most commonly lost tumour suppressors in human cancer. In order to understand the effect of these compounds on tumour suppressor proteins HepG2 cells were treated with compounds YM, A3 and A4 for 24 h and cell lysates were subjected to western analysis. An increase in the expression of both these proteins was seen upon treatment of the compounds (YM, A3 and A4) in comparison to control untreated cells indicating an effect in the upregulation of both the genes.

**Figure 5:**
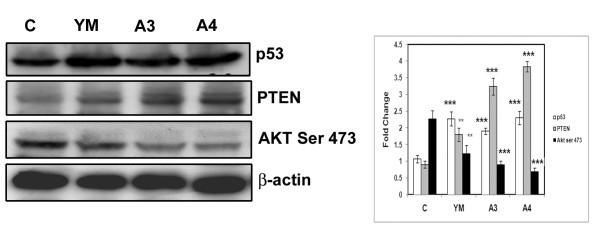
**Western blot analysis of tumour suppressor proteins (p53, PTEN) and PTEN dependent Akt phosphorylation in benzothiazole based compound treated cells**. HepG2 cells were treated with benzothiazole based compounds for 24 h and cell lysates were subjected to western blot analysis using antibodies specific to p53, PTEN and Akt Ser 473. β-actin was used as loading control. The molecular weights of p53, PTEN and Akt ser 473 are **53**, **47 **and **65 **KDa. The western blot experiments were repeated three times and densitometric analysis was carried out. The results were statistically significant with p-value <0.001 for A3 and A4 when compared to control.

**Figure 6:**
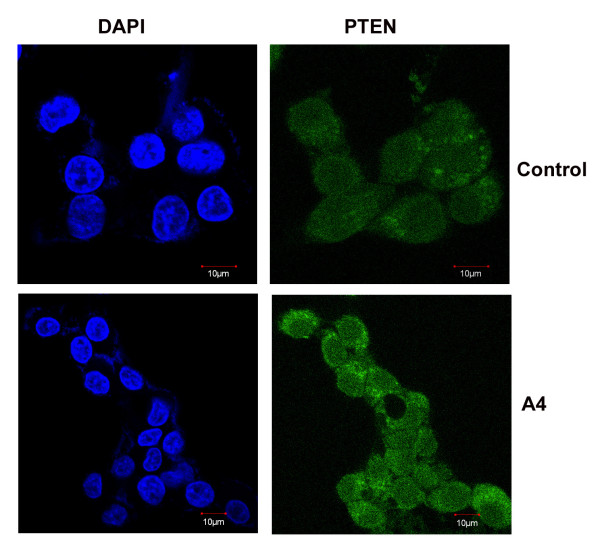
**Immunofluorescence based studies of PTEN after treatment with benzothiazole based compounds**. HepG2 cells were grown in 10% FBS containing medium in coverslips and treated with 4 μM of effective benzothiazole compound (A4). Cells were fixed and processed for immunofluorescence study as described in material and methods. Images were captured in confocal microscopy. The pattern of staining was also recorded. Control: represents untreated cells.

Apart from acting as tumour suppressor, PTEN can negatively regulate the activity of AKT through its lipid phosphatase function by dephosphorylating AKT at Ser473 [17]. As expected there was a significant reduction in the levels of phosphorlyated form of AKT (ser 473) in YM, A3 and A4 compound treated cells compared to untreated control cells. The expression of PTEN was further confirmed by carrying out immunofluoresence experiment in A4 (the effective compound) treated cells along with untreated control using PTEN antibody. A clear increase in the amount of fluorescence produced by staining with PTEN antibody in compound treated cells compared to untreated controls was observed (Figure 5 &6).

### Effect of Benzothiazole based compounds on Ras and MAP Kinase family proteins

**Figure 7:**
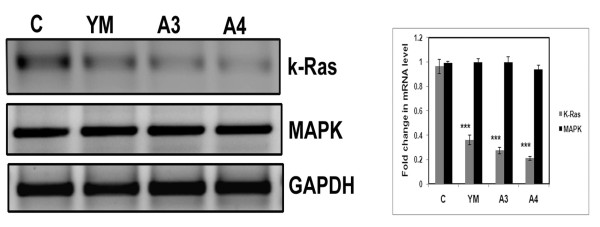
**Effect of benzothiazole based compounds k-Ras and MAPK transcript levels**. HepG2 cells were treated with benzothiazole based compounds (YM, A3 and A4) at a final concentration of 4 μM for 24 h. After treatment total RNA was isolated and RT-PCR was conducted. The PCR products were separated on 1% agarose gel electrophoresis and visualized under U.V. light. GAPDH was used as loading control. Each experiment was repeated three times. The gel pictures here were representative of three independent experiments and densitometric analysis was done. The results were found to be statistically significant when compared to control (p-value<0.001).

The activation of Ras is the first and foremost event in HCC [18] whose activation is known to regulate cell cycle progression through its interference with cyclins and cell cycle inhibitors. [19]. MAP kinase proteins constitute a serine threonine kinase protein family that decide the fate of cells either towards proliferation or differentiation [20,21]. Thus we checked the expression of k-Ras and p38 MAPK in untreated and compound (YM, A3 and A4) treated cells. Total RNA was isolated and RT-PCR was carried out using primers against Ras and p38MAPK genes. Drastic decrease in Ras mRNA level with no change in the levels of MAP Kinase transcript was observed in compound treated cells in comparison with untreated control cells (Figure 7).

**Figure 8:**
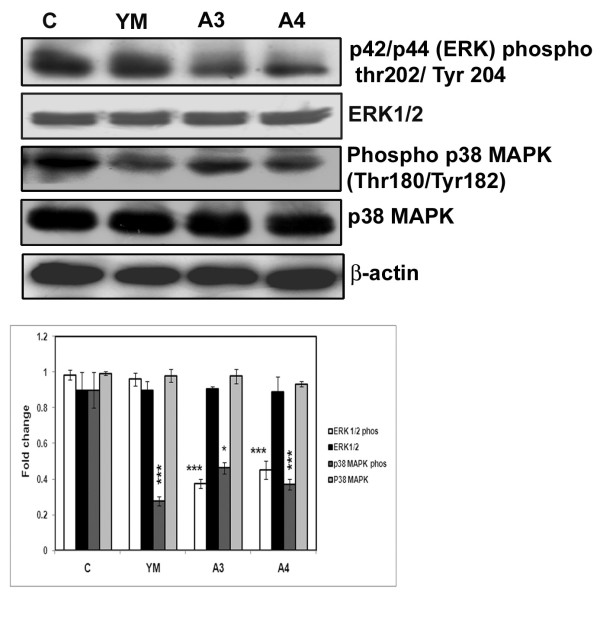
**Effect of benzothiazole based compounds on ERK and MAPK proteins that play vital role in cell proliferation**. HepG2 cells were treated with compounds YM, A3 and A4 for 24 h. The total lysates were harvested and analysed by immunoblotting with anti-ERK, phosphorylated ERK (Thr 202/Tyr 204), anti-p38 MAPK, phosphorylated form of p38 MAPK (Thr 180/Tyr 182) anti- bodies. YM serves as positive control. β - actin was used as loading control. Western blots were representative of three independent experiments. The expression of phosphorylated ERK and MAPK were found to be down regulated. C: represents control untreated cells. The molecular weights of ERK1/2, Phospho ERK1/2, p38MAPK and phosphop38MAPK were **42/44 **KDa, **42/44**KDa, **43 **KDa and **43 **KDa respectively. The western blot analysis was repeated thrice and densitometric analysis was done. The results were statistically significant with p < 0.001 for A3 and A4 with regard to ERK1/2 phosphorylation and p < 0.05, p < 0.001 for p38 MAPK phosphorylation for A3 and A4 respectively.

Previous studies have shown that proteins of MAPK family such as ERK1/2 and p38MAPK get activated upon phosphorylation and are responsible for rapid cell proliferation [22]. Therefore, we also quantitated the protein levels of p38 MAPK, ERK1/2 as well their active forms (*i.e *phosphorylated forms). Interestingly no change in the levels of MAPK and ERK1/2 was observed while the active phosphorylated forms were decreased revealing that these compounds have an inhibitory role on cell proliferation (Figure 8).

### Cytotoxicity induced by benzothiazole based compounds

**Figure 9:**
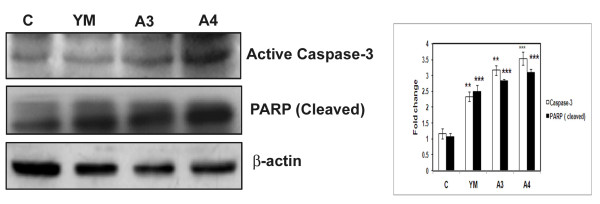
**Effect of benzothiazole based compounds in the activation of Caspase-3 and PARP**. HepG2 cells were treated with compounds YM, A3 and A4 for 24 h. The total cell lysates were harvested and analysed by immunoblotting with antibodies against active caspase-3 and cleaved PARP. YM is the positive control. β-actin was used as loading control. The results obtained were from three independent experiments and are statistically significant at p < 0.001 for compound A4.

The process of apoptosis is controlled by a diverse range of cell signals, which may originate either extracellularly (extrinsic) or intracellularly (intrinsic). To understand the mechanism underlying cell death we first studied the intrinsic pathway of apoptosis. HepG2 cells were treated with YM, A3 and A4 compounds at 4μM concentration for 24 h. Total cell lysates were obtained and western blot analysis was carried out using antibodies against active caspase-3 and cleaved PARP. An increased level of expression of both active caspase -3 and cleaved PARP in case of YM, A3 and A4 treated cells indicated the role of mitochondria in this apoptotic event via intrinsic pathway (Figure 9).

### Effect of benzothaizole based compounds on miR-195a and miR-101-1

**Figure 10:**
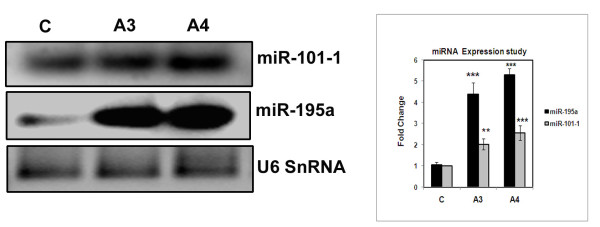
**Effect of benzothiazole based compounds on microRNAs miR-195a, miR-101-1**. The HepG2 cells were treated with compounds A3 and A4 at 4 μM concentration. Total RNA was isolated and the RNA was poly A tailed using Poly A polymerase and were subjected micro RNA specific RT-PCR study. Equal mRNA was used for the study. Each experiment was repeated three times. The results obtained were from three independent experiments and are statistically significant at p < 0.001 for compound A4.

Growing evidence indicate that deregulation of a class of non coding RNAs termed as microRNAs (miRNAs) contribute to tumourigenesis. Inhibition or alteration of miRNA expression in cancer cells impacts gene expression and affects cell proliferation and survival. Altered expression of miRNAs such as miR-101-1, miR-122a, miR-195, miR-21, miR-214, miR-373, miR-22, miR-221, miR-223 and miR-224 were observed in hepatocarcinoma. Recent studies have highlighted the importance of miR-195 that target cyclin D1 which regulate the G1-S phase transition, while ectopic expression of miR-101 dramatically suppressed the ability of hepatoma cells to form colonies [13,14]. Since both the compounds (A3, A4) have exhibited G1 cell cycle arrest, we were interested to check the expression of miR-195a and miR-101-1in the compound treated cells when compared to untreated control cells. HepG2 cells were treated with compounds A3 and A4 for 24 h at 4 μM concentration. The expression pattern of both the miRNAs was studied by Reverse transcription PCR (RT-PCR) analysis using specific primers. Surprisingly a 2- fold increase in the level of miR-101-1 and 5- fold increase in the level of miR-195a were observed in the case of effective (A4) compound treated cells when compared to untreated control cells. An almost similar result was observed in case of A3 treated cells. These results clearly show the regulatory role of benzothiazole compounds on cell cycle and apoptosis by modulating the non-coding RNAs that eventually lead to cell death (Figure 10).

## Discussion

Liver cancer is fatal and occurs commonly in Asian countries [23]. Hepato cellular carcinoma is characterised by rapid growth rate, strong malignancy, easy invasion, metastasis and poor prognosis. The development of chemotherapeutic agents that cause apoptosis and reduce mortality have been increasingly appreciated as ideal compounds for management of this cancer [24]. We have synthesised analogs of benzothiazoles (A3, A4) and carried out detailed biological study in order to understand the key signalling molecules that are affected and are responsible for the cytotoxic nature. In the present study benzothiazole conjuagates (A3 & A4) have shown profound cytotoxicity (50% of cell death) at 4μM concentration as observed by MTT based cell viability assay. The apoptosis inducing nature of the compounds was clearly demonstrated by Hoechst nuclear staining as well as tunnel assay which revealed blebbing and DNA fragmentation that occurred during cell death. The FACS analysis of A3 and A4 compound treated cells indicated G1 cell cycle arrest nature which was further confirmed by BrdU cell proliferation assay. The G1 to S phase transition of cell cycle is mainly controlled by proteins such as Cyclin D1 and Skp2. Perturbations in Cyclin D1 and its impact on hepatocarcinoma has been widely observed in 30 different hepatoma types [25]. Where as Skp2 plays a prominent role in the process of degradation of the tumour suppressor proteins such as Foxo that controls the G1 to S phase transition. In our study we found drastic down regulation of Cyclin D1and Skp2 proteins after compound treatment there by revealing G1 cell cycle arrest nature of the benzothiazole based compounds (Figure 4).

It has been reported that hepato-cellular carcinoma is the final event of inactivation of tumour suppressor genes such as PTEN and p53 that controls cell proliferation and death [7,8]. Upon compound treatment upregulation of PTEN and p53 protein levels was observed by western analysis. This data has been further supported by the fact that increased p53 level resulted in increased binding of p53 to PTEN promoter and controls both the transcriptional and translational regulation of PTEN gene which aids in increased rate of apoptosis [26].

Increased PTEN, apart from acting as tumour suppressor negatively regulates AKT activity (i.e AKT phosphorylation) and cause apoptosis [27]. Moreover AKT physically associates with MDM2 and phosphorylates at serine residue which helps in nuclear translocation thereby causing degradation of p53 [28]. In the present study we observed increased PTEN which causes decreased AKT activity (phosphorylation) and finally leading to p53 activation. Another important protein family that plays a major role in the formation, migration, invasion and maintainence of hepatocarcinoma is MAPK protein family. Mitogen activated protein kinase (MAPK) family members such as MAPK and ERK1/2 are important proteins that functions as intracellular signal transducers which enter into the nucleus upon phosphorylation [22] and induce genes involved in the process of proliferation and differentiation [22] &[21]. Interestingly treatment of HepG2 cells with the compounds (A3 & A4) resulted in the decrease of phosphorylated forms of P38 MAPK and ERK1/2 proteins thus controlling active cell proliferation.

Recent studies have indicated that micro RNAs are directly involved in many different kinds of cancers such as lung, breast, brain, liver, colon and leukemia. More than 50% of miRNA genes are located in cancer associated genomic regions or in the fragile sites suggesting that miRNAs may play a vital role in the pathogenesis of human cancers [29]. Interestingly degradation of microRNAs has been frequently observed in tumour tissues [30,31]. In 80-90% of hepato cellular carcinoma (HCC) cases microRNAs such as miR-195 and miR-101-1 were found to be down regulated. Ectopic expression studies of miR-195 caused G1/S phase block by targeting cyclin D1 [13] whereas miR-101-1 dramatically suppresses the ability of hepatoma cells to form colonies and develop tumours [14]. Upon compound treatment (A3 and A4) the expression of miRNAs (miR-195, miR-101-1) was significantly increased and thus has potential implication in the treatment of hepatoma. Previous studies [32-34] have shown the role of caspase-3 and cleavage of PARP in the process of apoptosis. The benzothiazole compounds in the present study have also shown the involvement of mitochondria through the activation of caspase-3 and PARP proteins.

## Conclusions

Benzothiazole based compounds (A3, A4) exhibited high cytotoxic activity as observed from the MTT studies. Cell cycle analysis has shown the G1 cell cycle arrest nature of the compounds and was further confirmed by BrdU cell proliferation assay. Our compounds A3 and A4 regulate cell cycle by affecting cyclin D1 and Skp2 that play a vital in controlling G1-S phase of cell cycle. The balance between genes involved in cell proliferation and tumour suppression regulate the events of tumourigenesis or apoptosis. Compound treated cells have exhibited increased levels of tumour suppressor proteins PTEN and p53. The phosphorylated (active) forms of ERK1/2 and p38 MAPK protein levels were decreased in compound treated cells which led to control of cell proliferation. The levels of microRNAs such as miR-195a, miR-101-1 were elevated in A3 and A4 compound treated cells compared to control untreated cells. These compounds caused cell death involving mitochondria as the major organelle with the activation of active caspase-3 and PARP proteins. Thus these benzothiazole based conjugates have potential implications in control of hepato-cellular carcinoma.

## Methods

### Cell culture

Human hepato-carcinoma cell line HepG2 purchased from American Type culture collection was maintained in Dulbecco's modified Eagle's medium (DMEM) (Invitrogen), supplemented with 2 mM glutamax (Invitrogen), 10% fetal calf serum and 100 U/ml Pencillin and 100 mg/ml streptomycin sulfate (Sigma). The cell line was maintained at 37°C in a humidified atmosphere containing 5% CO_2 _in the incubator.

### MTT assay

Cell viability was assessed by MTT assay, a mitochondrial function assay. It is based on the ability of viable cells to reduce the MTT to insoluble formazan crystals by mitochondrial dehydrogenase. HepG2 cells were seeded in a 96-well plate at a density of 10,000 cells/well. After overnight incubation, cells were treated with compounds **YM**, **A3 **and **A4 **at 4 and 8 mM concentration and incubated for 24 h. Medium was then discarded and replaced with 10 μL MTT dye. Plates were incubated at 37°C for 2 h. The resulting formazan crystals were solubilized in 100 μL extraction buffer. The optical density (O.D) was read at 570 nm with micro plate reader (Multi-mode Varioskan instrument-Themo Scientific).

### Cell Cycle Analysis

5 × 10^5 ^HepG2 cells were seeded in 60 mm dish and were allowed to grow for 24 h. Compounds **A3**, **A4 **and **YM **were added at a final concentration of 4μM to the culture media, and the cells were incubated for an additional 24 h. Cells were harvested with Trypsin-EDTA, fixed with ice-cold 70% ethanol at 4°C for 30 min, washed with PBS and incubated with 1 mg/ml RNase A solution (Sigma) at 37°C for 30 min. Cells were collected by centrifugation at 2000 rpm for 5 min and further stained with 250 mL of DNA staining solution [10 mg of Propidium Iodide (PI), 0.1 mg of trisodium citrate, and 0.03 mL of Triton X-100 were dissolved in 100 mL of sterile MilliQ water at room temperature for 30 min in the dark]. The DNA contents of 20,000 events were measured by flow cytometer (DAKO CYTOMATION, Beckman Coulter, Brea, CA). Histograms were analyzed using Summit Software.

### Tunel assay

Tunel assay was conducted by using the Apoalert DNA fragmentation Assay kit (Clone tech) according to manufacturer instructions. This kit detects the apoptosis-induced nuclear DNA fragmentation that is based on fluorescence. The assay is based on the principle of terminal deoxy nucleotidyl transferase (TdT)-mediated dUTP nick-end-labelling (*i.e *TUNEL). TdT catalyzes incorporation of fluorescein-dUTP at the free 3'-hydroxyl ends of fragmented DNA. Flourescein-labeled DNA can be detected via confocal microscopy.

### BrdU Cell Proliferation Assay

This assay was carried out by using the 5-Bromo 2-deoxyuridine (BrdU) cell proliferation assay kit (Millipore) to assess the effect of YM, A3 and A4 compounds on the proliferation of HepG2 cells. 1 × 10^4^cells were seeded and allowed to grow for 24 h. BrdU was added and allowed to incorporate for 5 h followed by the addition of test compound (YM, A3 & A4) at concentration of 4 μM for 24 h. Fixation was done for 30 min at room temperature. The cells were then washed, anti-BrdU antibody was added which binds to BrdU that was incorporated in the cell. After 1 h incubation, 100 μl anti-BrdU goat anti-mouse horse raddish peroxidise (HRP)- conjugated secondary antibody (1:2000) was added and incubated for 30min. Washing procedures were followed according to the manufacturer's instructions. TMB substrate (100 μl) was added, incubated for another 30min at room temperature and O.D values were taken at a wave length of 450nm. Lower O.D values reflect lower BrdU concentrations in the sample and thus an indirect depiction of a low cell proliferation rate.

### Immunofluorescence

Human hepato-carcinoma HepG2 cells were seeded on cover slips and treated with benzo-thiazole based compounds at concentration of 4 μM for 24h. After treatment, cover slips were fixed with a paraformaldehyde solution (4% in 1× PBS) for 20 min at room temperature. Cell permeabilization was achieved by administration of a Triton X-100 solution (0.2% in 1× PBS) for 5min. Further the cover slips were kept overnight in 100% methanol at 4°C. Subsequently, cover slips were blocked with a 1% BSA solution for 60min and then incubated with anti PTEN (1:100) antibody at room temperature for 2h. The slides were washed three times each of 5min with PBST. Then cover slips were incubated with a FITC-conjugated anti-rabbit secondary antibody (Jackson Immuno Research Laboratories Inc., Pennsylvania, USA) for one hour and cover slips were washed three times with PBST solution and mounted with DAPI/PI solution. Finally, cells were observed under confocal microscope (Olympus FV1000). Images taken were processed with the support of the flow view version 1.7 c software program.

### Protein extraction and Western blot analysis

Total cell lysates from cultured HepG2 cells were obtained by lysing the cells in ice-cold RIPA buffer (1XPBS, 1% NP-40, 0.5% sodium deoxycholate and 0.1% SDS) and containing 100 mg/mL PMSF, 5 mg/mL Aprotinin, 5 mg/mL leupeptin, 5 mg/mL pepstatin and 100 mg/mL NaF. After centrifugation at 12,000 rpm for 10 min, the protein in supernatant was quantified by Bradford method (BIO-RAD) using Multimode Varioskan instrument (Thermo-Fischer Scientifics). Fifty micrograms of protein per lane was applied in 12% SDS-polyacrylamide gel. After electrophoresis, the protein was transferred to polyvinylidine difluoride (PVDF) membrane (GE Biosciences). The membrane was blocked at room temperature for 2 h in TBS + 0.1% Tween20 (TBST) containing 5% blocking powder (Santacruz). The membrane was washed with TBST for 5 min, primary antibody was added and incubated at 4°C overnight (O/N). ERK 1/2, ERK Phos (Thr 202/Tyr 204), Phos p38 (Thr 180/Tyr 182), P38MAPK, active caspase-3 and Cleaved PARP antibodies were purchased from Cell Signalling, USA. P53 and Cyclin D1antibodies were purchased from Santa Cruz, PTEN and Skp2 antibodies were purchased from Abbiotec Company. Akt Ser 473 antibody was purchased from (Millipore). The membrane was incubated with corresponding horseradish peroxidase-labeled secondary antibody (1:2000) (Santa Cruz) at room temperature for 1h. Membranes were washed with TBST three times for 15 min and the blots were visualized with chemiluminescence reagent (Thermo Fischer Scientifics Ltd.). The X-ray films were developed with developer and fixed with fixer solution.

### Nuclear staining

HepG2 cells were seeded on cover slips, treated with compounds for 24h, washed with PBS and fixed with 4% Paraformaldehyde for 15min at room temperature. Fixed cells were incubated in PBS (pH 7.4) containing DNAse-free RNase (Sigma) for 30 min at 37°C and stained with Hoechst. Nuclear morphology of the cells was observed under confocal microscope.

### Semi-quantitative Reverse Transcription PCR (RT-PCR)

Total RNA was extracted using RNeasy mini kit (Qiagen, USA) and reverse transcribed into cDNA using superscript II reverse transcriptase (Invitrogen life technologies). The PCR was carried out with specific primers such as K-Ras, MAPK (Table 1) in Takara Bioscience PCR machine. The products were electrophoresed on agarose gel (1%) followed by staining with ethidium bromide and visualized under U.V. light. The signal intensity of respective bands was measured by means of the quantity one version 4.1.1 soft ware using BIORAD image analysis system (CA, USA).

**Table 1:** List of primer sequences used for RT-PCR

Primer	Sequence	Product size (bp)
K Ras -FP	5'-tacagtgcaatgagggacca-3'	206
K Ras -RP	5'-tcctgagcctgttttgtgtct-3'	
MAPK- FP	5'-ccagaccatgatcacacagg-3'	163
MAPK-RP	5'-ctggaaagatgggcctgtta-3'	

### MicroRNA Expression profiling

Total RNA was isolated from control and compound (A3 and A4) treated cells. Equal amount of DNase-treated RNA was Poly A tailed using Poly A Polymerase and oligo dT adapter was used to synthesise the cDNA. RT-PCR reaction was set up using universal reverse primer and miRNA specific forward primer. The temperature conditions are 50°C for 2min, 95°C 10min, followed by 40 cycles of (95°C 15sec, 60°C 1min).

### Statistical Analysis

Statistical Analysis was performed using the graph pad software to evaluate the significant difference between the control and treated samples. All variables were tested in three independent experiments. The results were reported as mean ± SD. * indicates p < 0.05, ** indicates p < 0.01 and *** indicates p < 0.001.

## Abbreviations

HCC: Hepato-cellular carcinoma; ERK: Extracellular signal-regulated kinases; MAPK: Mitogen-activated protein kinase; miRNA: micro RNA; PTEN: Phosphatase and tensin homolog; YM: YM-201627.

## Competing interests

The authors declare that they have no competing interests.

## Authors' contributions

SNCVLPV, MJR designed the experiments and performed the miRNA profiling, nuclear staining and WB. CS, DM, JLA performed RT-PCR, Immunofluorescence and cell culture experiments. RK synthesized the compounds. UB and MPB conceived the idea, analyzed the data and prepared the manuscript. All authors read and approved the final manuscript.
